# Oral Health Trends and Challenges in North and West Africa: A Systematic Review of Cross-Sectional Studies

**DOI:** 10.3390/healthcare14060821

**Published:** 2026-03-23

**Authors:** Rocío Trinidad Velázquez-Cayón, Juliana Cassol Spanemberg, Ana del Toro Arencibia, Elena Sirumal, Susell Parra-Rojas

**Affiliations:** 1 Oral Health in Vulnerable Patients Research Group—VULPARE, Faculty of Health Sciences, Fernando Pessoa Canary Islands University, 35450 Gran Canaria, Spain; jcassol@ufpcanarias.es (J.C.S.); esirumal@ufpcanarias.es (E.S.); sparra@ufpcanarias.es (S.P.-R.); 2 Department of Dentistry, Faculty of Health Sciences, Fernando Pessoa Canary Islands University, 35450 Gran Canaria, Spain; 3 Department of Psychology, Faculty of Health Sciences, Fernando Pessoa Canary Islands University, 35450 Gran Canaria, Spain

**Keywords:** oral health, North Africa, West Africa, dental caries, periodontal diseases, socioeconomic determinants, systematic review

## Abstract

**Highlights:**

**What are the implications of the main findings?**

- There is a critical need to transition from current symptom-driven, extraction-oriented dental care models toward integrated, primary care systems focused on prevention and tooth-preserving interventions.
- Future public health policies must prioritize the standardization of epidemiological reporting and the inclusion of patient-centered metrics to effectively address structural inequalities and reduce the oral health burden in the region.

**Abstract:**

Background/Objectives: Oral diseases represent a major public health challenge in Africa, considering socioeconomic disparities and limited healthcare access. This systematic review aimed to comprehensively analyze the oral health status, conditions, and associated socioeconomic and cultural associated factors in North and West African regions. Methods: A systematic search was conducted in PubMed, Web of Science, and Scopus for cross-sectional studies. Using the CoCoPop framework, 19 studies were selected and evaluated for risk of bias using the JBI critical appraisal tool. Results: The findings reveal a substantial burden of untreated pathology, with localized caries prevalence reaching 74% in children. Periodontal health is consistently compromised in adults, characterized by high levels of calculus and gingival bleeding. Self-reported data highlight a symptom-driven culture, where lower-socioeconomic-status (SES) households rely on traditional remedies or emergency extractions due to economic and geographic barriers. Conclusions: Oral health in North and West Africa is characterized by profound inequalities. Current systems fail to reach vulnerable populations, emphasizing the urgent need for a structural shift toward integrated, equity-oriented primary care models that prioritize prevention over reactive, extraction-based treatments.

## 1. Introduction

The World Health Organization (WHO) emphasizes the importance of oral health, as it not only contributes to the functional capacity of mastication and phonation but also constitutes an essential component of overall health and well-being [1]. Moreover, the implementation of appropriate preventive and therapeutic measures to address oral diseases is a determining factor, since it can prevent serious complications such as systemic infections, cardiovascular conditions, and potential complications during pregnancy [2]. Consequently, the promotion of oral health emerges as a priority within the field of public health. Currently, oral diseases pose a global public health challenge, as the high incidence of oral diseases contributes to a negative impact on individual’s quality of life [1,2,3,4]. The concept of oral health-related quality of life involves considering not only physical aspects such as pain or functional changes but also emotional and social dimensions associated with oral health. Its direct influence on quality of life is noteworthy, as it may lead to functional, aesthetic, nutritional, and psychological problems [5,6].

Africa exhibits profound disparities across its population segments in wealth levels, overall well-being, healthcare accessibility, and educational achievements. These contrasts are mirrored in the substantial economic heterogeneity across African nations, characterized by stark economic and tribal divides, alongside sparsely distributed rural communities juxtaposed against densely populated urban centers [7,8].

Widespread poverty remains a major challenge across many African countries, where high levels of undernutrition coexist with a substantial burden of endemic and water borne infectious diseases. Limited access to safely managed drinking water, inadequate sanitation and poor hygiene practices, including oral hygiene, further amplify the risk of preventable morbidity and mortality. These conditions are frequently aggravated by weak governance and episodes of political instability, which undermine health systems, disrupt education and constrain investments in basic infrastructure. Addressing these challenges requires integrated and collaborative, multisectoral strategies that simultaneously target poverty reduction, nutrition, water and sanitation, and health system strengthening to improve living conditions and population health in African settings [9,10].

The United Nations has utilized this division of Africa into geographical regions for statistical and organizational purposes since the creation of the United Nations Statistics Division (UNSD) in 1947. This classification has been maintained and periodically updated since then. The latest update to the United Nations geographical region map for Africa, which includes the division into regions such as Northern Africa, Western Africa, Eastern Africa, Central Africa, and Southern Africa, was carried out in 1999. It is responsible for collecting, processing, and disseminating statistical information for the UN. This geographical region map, created and updated in 2024, serves purposes of statistical analysis [11].

West Africa is widely recognized as one of the world’s poorest regions, concentrating a very high share of the global multidimensionally poor population despite representing only a fraction of the world’s inhabitants. Analyses based on the Global Multidimensional Poverty Index show that more than half of people in African countries are multidimensionally poor, with particularly high headcount ratios in West African settings, and that the vast majority of these poor individuals live in rural areas [9,10].

Although oral diseases are not commonly fatal, they represent a major public health problem due to their high incidence and significant impact on overall health. These conditions result from various modifiable risk factors common to many non-communicable diseases (NCDs), such as sugar consumption, tobacco and alcohol use, and poor oral hygiene, along with their underlying social and economic associated factors [12,13,14,15].

The WHO 2022 Global Oral Health Report estimates that approximately 3.5 billion people worldwide suffer from oral diseases, with three-quarters residing in middle-income countries. The prevalence of major oral diseases continues to rise globally due to increasing urbanization and changes in living conditions, primarily driven by lack of fluoride exposure, the availability and accessibility of sugar-rich foods, and limited access to community oral care services [1].

Dental caries is the most prevalent oral disorder, with observed increases in incidence across numerous African countries in recent years. Recent epidemiological studies in developing countries suggest that the prevalence and severity of dental caries have risen with industrialization and the adoption of Western diets. Income levels can indirectly influence caries susceptibility, as lack of economic resources, often linked to lower education levels and harmful health habits, as well as limited access to healthcare information, correlates with higher caries incidence. Additionally, low parental education can hinder access to and understanding of oral disease prevention importance [15,16].

To provide a comprehensive overview of oral health in North and West Africa, the following specific objectives were established:

1. To determine the prevalence and severity of the most common oral conditions—dental caries, periodontal diseases, and tooth loss—among different age groups in the country.
2. To analyze the association between oral health status and socioeconomic, behavioral, and contextual associated factors, such as education, urban–rural residence, and oral hygiene practices.

## 2. Materials and Methods

### 2.1. Protocol Recording

The review was conducted in accordance with the PRISMA (Preferred Reporting Items for Systematic Reviews and Meta-Analyses) guidelines [17] (Supplementary File S1: PRISMA checklist), and the study protocol has been registered in the PROSPERO database under identification number CRD420261283702.

### 2.2. Focused Question

“What is the oral health status in North and West Africa, and what are the most prevalent oral health conditions in this region?”

### 2.3. Eligibility Criteria

In this systematic review, the following inclusion and exclusion criteria were established, with included studies organized into two categories for presenting results: cross-sectional descriptive studies describing the most prevalent oral health conditions through intraoral and extraoral examinations and their relationship with socioeconomic and cultural factors, and studies evaluating or complementing oral health status information through self-perception surveys.

Among the exclusion criteria are studies analyzing and/or comparing different oral health plans in North and West African countries, systematic reviews, editorials, letters to the editor, studies linking oral health with other systemic pathologies, studies specific to pregnant women, studies on institutionalized individuals such as those in orphanages or prisons, studies on cohorts in very isolated locations, studies analyzing the oral health status of migrant or refugee individuals in the host country, studies with samples consisting of health professionals or health science students, and studies focusing on evaluating the population’s level of knowledge regarding oral health.

### 2.4. The CoCoPop Strategy

The CoCoPop strategy was selected [18] as it is more appropriate for prevalence and population-level observational studies, allowing a comprehensive identification of relevant evidence by clearly defining the health condition of interest (Condition), the study setting or environment (Context), and the target population (Population):

- Condition: Oral health outcomes included in the review.
- Context: African countries included in the review.
- Population: Adults, adolescents and children.

### 2.5. Information Sources and Search Strategy

The bibliographic search, conducted in October 2025, encompassed databases such as PubMed, Web of Science and Scopus. Employing Medical Subject Headings (MeSH) in combination with Boolean operators “AND” and “OR”, a tailored search strategy was adapted for each database (Supplementary File S2: Database search strategy).

### 2.6. Data Collection Processing and Data Items

Data collection was performed by two independent reviewers (A.T.A. and R.T.V.C.), using a piloted extraction form developed from the review objectives and CoCoPop framework, with a third reviewer (S.P.R) resolving discrepancies. The Kappa concordance test yielded a coefficient of 0.80, indicating considerable agreement and reinforcing the reliability of the study selection process. For each eligible study, data were extracted and organized into a standardized table covering: bibliographic information (author, year), study design, setting/country, population characteristics (age range, sample size), measurement instruments, clinical and self-reported outcomes, and socioeconomic status (SES) indicators. Clinical indices included the Decayed, Missing, and Filled Teeth index (dmft /DMFT), Community Periodontal Index of Treatment Needs (CPITN/CPI), and Oral Hygiene Index-Simplified (OHI-S).

In addition, information was collected on the main oral conditions and outcomes reported (prevalence and/or severity of dental caries, periodontal conditions, tooth loss and other relevant oral pathologies), indicators of socioeconomic position (education, occupation, asset based indices, social class, urban–rural residence) and behavioral or contextual variables (toothbrushing practices, use of fluoridated toothpaste, sugar consumption, tobacco use, dental attendance and type of provider). When available, measures of association between oral health outcomes and socioeconomic or behavioral factors, as well as key conclusions related to inequalities in oral health, were also recorded to facilitate the subsequent narrative synthesis.

### 2.7. Study Risk of Bias Assessment 

The methodological quality of the studies followed the Joanna Briggs Institute (JBI) critical evaluation tool for checklist for cross sectional studies [18]. The following critical evaluation criteria were considered:

1. Were the criteria for inclusion in the sample clearly defined?
2. Were the study subjects and the setting described in detail?
3. Was the exposure measured in a valid and reliable way?
4. Were objective, standard criteria used for measurement of the condition?
5. Were confounding factors identified?
6. Were strategies to deal with confounding factors stated?
7. Were the outcomes measured in a valid and reliable way?
8. Was appropriate statistical analysis used?

Each study was independently assessed. For synthesis, an overall risk of bias category was assigned according to the pattern of JBI responses: studies with “yes” on most key items (particularly clear inclusion criteria, valid and reliable exposure and outcome measures, explicit identification and statistical control of confounders, and appropriate analyses) were classified as low risk of bias; studies with some concerns (for example, incomplete reporting or partial handling of confounding or measurement issues) were classified as moderate risk; and studies with multiple “no” or “unclear” ratings on core domains were classified as high risk of bias.

### 2.8. Effect Measures

Given the descriptive, cross-sectional design, effect measures focused on quantifying disease burden and characterizing the relationship between oral health and contextual factors.

Oral health status and the most prevalent conditions were described using clinical metrics such as the frequency and severity of dental caries (measured by dmft/DMFT/deft indices), periodontal conditions (including bleeding on probing, calculus, and periodontal pockets categorized via CPITN or CPI), and tooth loss (quantified by the number of missing teeth or the prevalence of partial and complete edentulism).

The relationship between oral health status and socioeconomic, behavioral, or contextual variables was described through measures of association reported by the original studies. This included comparisons across categories of education, parental occupation, asset-based indices, and urban–rural residence, as well as oral health behaviors (e.g., toothbrushing frequency, sugar consumption) and care-seeking patterns. Due to the substantial heterogeneity in study populations, age groups, and diagnostic criteria, these measures were synthesized narratively and organized by life stage (children, adolescents, and adults) and type of outcome (clinical versus self-perceived) to ensure clinical relevance.

### 2.9. Synthesis Methods

A narrative synthesis of the selected studies was conducted. The qualitative synthesis was conducted in a structured manner using the study characteristics and outcomes summarized in the extraction table. First, the included studies were grouped according to the two prespecified categories: clinical findings and disease burden and self-reported oral health and behaviors.

Due to the significant heterogeneity observed in study populations, age groups, and diagnostic criteria across the included studies, findings were structured and compared by life stage (categorizing results into children, adolescents, and adults) to ensure clinical relevance and facilitate the identification of age-specific oral health patterns in the region.

## 3. Results

### 3.1. Literature Search and Selection

A total of 3350 records were identified through database searches: 995 from PubMed, 936 from Scopus, and 1419 from Web of Science. After removing 1168 duplicate records, 2182 unique titles and abstracts were screened, resulting in the exclusion of 1719 records that did not meet the initial eligibility criteria.

**Figure 1:**
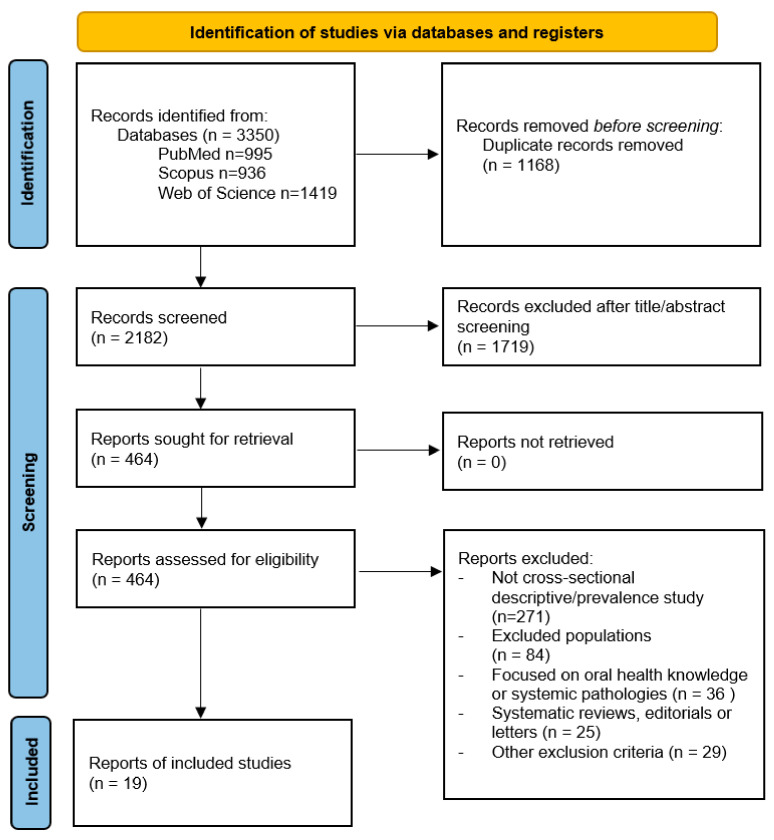
The flowchart of studies included.

Of the 464 reports sought for retrieval, all were successfully retrieved and assessed for eligibility. During this phase, 445 reports were excluded for the following reasons: 271 were not cross-sectional descriptive or prevalence studies; 84 involved excluded populations; 36 focused on oral health knowledge or systemic pathologies; 25 were systematic reviews, editorials, or letters; and 29 met other specific exclusion criteria. Following this rigorous selection process, a total of 19 studies were included in the systematic review (Figure 1).

### 3.2. Characteristics of Included Studies

The 19 included cross-sectional studies (Table 1) were published between 1999 and 2023 [19,20,21,22,23,24,25,26,27,28,29,30,31,32,33,34,35,36,37], providing a comprehensive overview of oral health trends over more than two decades in North and West Africa. The geographical distribution encompassed several low- and middle-income countries, specifically Algeria, Burkina Faso, Egypt, Ghana, Niger, Nigeria, and Tunisia.

Most investigations were school-based surveys focusing on the pediatric and adolescent population. Others utilized community-based population surveys or household-based cross-sectional designs to assess adults and families in their natural living environments. A smaller but significant proportion of the evidence was derived from secondary analyses of national data, such as the WHO STEPwise approach to surveillance (STEPS) and national oral health surveys. Additionally, clinic-based series were employed to analyze specific treatment-seeking patterns, while national pathfinder surveys were used to provide a broad diagnostic overview of oral health status. Sample sizes showed significant heterogeneity, reflecting both local and national scopes. These ranged from smaller, localized cohorts, such as 369 Egyptian children and 496 Nigerian adults, to large-scale national representative samples, including 4677 Burkinabe adults, 5954 Egyptian adults, and 6989 Algerian adults.

Regarding assessment methodologies, oral health conditions were predominantly measured using standard clinical indices established by the World Health Organization (WHO). The dmft/DMFT was the primary measure for dental caries, while periodontal status was assessed through the Community Periodontal Index of Treatment Needs (CPITN/CPI) and oral hygiene through the Simplified Oral Hygiene Index (OHI-S).

The primary exposures and contextual factors analyzed across the studies included socioeconomic position (attained education, parental occupation, and urban–rural residence), health behaviors (frequency of toothbrushing, use of fluoridated toothpaste, and tobacco use), and healthcare-seeking patterns, frequently highlighting a predominance of emergency-driven, extraction-oriented care over preventive dental visits.

### 3.3. Clinical Findings and Disease Burden

In studies based on clinical examination [19,20,21,22,23,25,26,27,28,29,30,32,33,35,36], caries experience in children showed a wide range of prevalence, with a substantially higher burden in the primary dentition compared to low-to-moderate mean dmft/DMFT scores in the permanent teeth. National and local surveys revealed that approximately half of the schoolchildren were affected by dental caries, with a high proportion of lesions remaining untreated. Specifically, localized studies in Egypt and Nigeria reported caries prevalence rates of 74% and 24.6% among children and adolescents, respectively, reflecting significant regional variations.

Across adolescent and adult populations, clinical data consistently demonstrated a combination of untreated caries, high levels of calculus, and gingival bleeding. There was a considerable prevalence of periodontitis and clinical attachment loss, particularly among older adults. Furthermore, clinic-based samples highlighted a clear predominance of symptom-driven, extraction-oriented care, indicating a lack of preventive or restorative focus. These clinical patterns were documented in both urban and rural settings and were frequently associated with a limited oral health workforce and poor service availability, underscoring substantial unmet treatment needs in the region.

### 3.4. Self-Reported Oral Health and Behaviors

In studies primarily based on self-reported measures [20,24,31,34,37], a significant proportion of adolescents and adults rated their oral health as poor or very poor.

Regarding oral hygiene practices, while daily toothbrushing was common, the frequency and quality of these habits varied. Participants frequently reported chewing difficulties and impaired. For instance, although 69.1% of adults in certain West African urban settings reported brushing twice daily, consistent use of fluoridated toothpaste and regular dental attendance remained infrequent across the region. In children, hygiene habits were strongly influenced by parental education and SES; lower parental education was directly associated with decreased brushing frequency and a higher burden of caries. Furthermore, a notable reliance on traditional cleaning methods persists, such as the use of chewing sticks (miswak), which are still preferred by a sizeable portion of the population for their perceived antibacterial properties and cultural relevance.

Self-reported data on healthcare-seeking patterns further revealed profound inequalities. Households with lower-SES were more likely to rely on home remedies, traditional healers, or non-dentist providers, often resulting in extractions as the primary solution for advanced caries. Conversely, more advantaged groups reported higher attendance at private dental clinics and access to a broader range of restorative treatment options. These disparities underline a clear social gradient where education, income, and urban–rural residence act as major associated factors of oral health outcomes in North and West Africa.

### 3.5. Socioeconomic Determinants and Oral Health Inequalities

Clinical patterns of untreated caries and periodontal disease were documented in both urban and rural settings, yet they were frequently exacerbated in contexts of limited oral health workforce and service scarcity, as noted in studies from Nigeria and Burkina Faso [25,34].

Social gradients were clearly visible through indicators such as education, income, and urban–rural residence [30,33]. In children, lower parental education was a significant predictor of higher caries prevalence and poorer hygiene habits [30]. Furthermore, self-reported data on care-seeking revealed that lower-SES households were more likely to rely on traditional remedies, such as chewing sticks or “miswak”, non-dentist providers, or emergency extractions to manage pain [25,36]. In contrast, more advantaged groups reported more frequent attendance at private dental clinics and access to a wider range of tooth-preserving treatment options [27,28]. These findings underscore substantial unmet treatment needs and the persistent challenge of shifting from emergency-driven models to preventive, equity-oriented care in the region.

### 3.6. Risk of Bias Assessment

Identification and handling of confounding factors (items 5 and 6) was the main methodological limitation identified across the included studies. Recent national surveys and studies employing robust multivariable analyses, such as Hewlett et al. 2022 [36], Aly et al. 2020 [30], Diendéré et al. 2022 [34], Abou El Fadl et al. 2021 [33], Uguru et al. 2021 [31], Pengpid et al. 2023 [37], and Varenne et al. 2011 [26], explicitly pre-specified sociodemographic and behavioral confounders and adjusted for them, leading to a Yes rating on both items and an overall low risk of bias (Table 2).

In contrast, older descriptive surveys, clinic-based series, or studies focusing primarily on bivariate correlations—including Agbelusi et al. 2007 [25], Abbass et al. 2019 [29], Abid et al. 2004 [22], Bruce et al. 2002 [21], Varenne et al. 2005 [23], Petersen et al. 1999 et al. [19], and Ojofeitimi et al. 2007 [24]—often reported sociodemographic variables without formally defining them as confounders or systematically adjusting for them in the final analysis. Consequently, these were rated Unclear for items 5 and 6 and judged to have a moderate risk of bias. Statistical analyses (item 8) were generally appropriate across the board, as most studies utilized regression models or stratified analyses aligned with their specific objectives (Table 2).

## 4. Discussion

This systematic review aimed to comprehensively analyze the oral health status across North and West African regions by identifying the most prevalent oral diseases and their associated socioeconomic and cultural factors. In addition, it sought to examine the main barriers limiting access to oral healthcare services, the available resources for prevention and treatment, and the methodological approaches adopted in studies conducted within these settings. Our findings, derived from 19 cross-sectional studies published between 1999 and 2023 (no eligible studies were identified for 2024–2025), reveal a substantial burden of unmet dental needs and structural inequalities.

In studies based on clinical examination, a substantial burden of oral disease was evident from early ages, characterized primarily by high proportions of dental caries in the primary dentition, with prevalence reaching 74% in localized samples, and a low therapeutic index that leaves many lesions untreated in both dentitions. Furthermore, periodontal health was consistently compromised across adolescents and adults, who exhibited frequent calculus deposits, gingival bleeding, and clinical attachment loss, alongside a considerable prevalence of tooth loss and a predominance of symptom-driven, extraction-focused treatment patterns. Across several settings, the distribution of these oral conditions was closely linked to structural and social disadvantage, including rural poverty and marked imbalances in the oral health workforce.

Regarding self-reported oral health and behaviors, the evidence highlighted recurring patterns of poor self-rated oral health and impaired quality of life, particularly among older adults. Although basic daily hygiene was commonly reported, the recommended twice-daily brushing and the consistent use of fluoridated toothpaste remained infrequent, showing clear social gradients by education and income. Moreover, recent dental attendance was uncommon due to economic and geographic barriers, leading lower-SES households to rely frequently on traditional remedies, such as chewing sticks (miswak), or non-dental providers to manage caries episodes. These findings underscore a persistent challenge in shifting from emergency-driven responses toward preventive and equity-oriented models of care in the region.

An international web-based survey by John MK et al. [38] involving 1580 dentists reinforced that effective interventions should be evaluated not only on clinical indicators but also on their impact on function, pain, aesthetics, and psychosocial well-being. The assessment of oral health-related quality of life has established itself as a valid tool for diagnosis and treatment planning, allowing for the prediction of patient adherence based on individual perceptions and expectations [39].

The scoping review by Carrasco-Labra et al. [40] examined the barriers and facilitators for oral health policies in the WHO African region, emphasizing that understanding these factors is crucial for improving the efficiency of health systems and the quality of health outcomes. Access to oral health services varies considerably between countries and is largely demand-driven rather than a result of rigorous planning. Globally, oral health care is primarily provided by private professionals, covering only a small portion of health needs [41,42]. In low-income countries, professionals tend to concentrate in urban areas, leaving a significant part of the population with limited access to primary care. Cost remains a major obstacle to accessing these services [43].

Health programs play a crucial role in population well-being. Ghotane et al. [44] conducted a program in Sierra Leone that revealed a lack of qualified personnel, estimating a need for 27 to 163 dental therapists to satisfy population requirements. Adeniyi et al. [45] examined the Nigerian oral health system, demonstrating that it did not respond effectively to population needs due to poor management. Furthermore, Folayan et al. [46] addressed the prevalence and consequences of dental caries in Nigerian children, finding that limited access due to economic and structural barriers resulted in low service utilization.

Only 54% of the African population utilizes dental and preventive services [11]. Due to the lack of resources and medical facilities, the population has minimal access to oral care, leading to high levels of preventable diseases [13,47]. Understanding the situation in their home countries is essential to evaluate how oral health conditions interfere with the quality of life of individuals in a continuous migration environment [40]. The urban–rural asymmetry and high costs associated with dental care represent significant barriers, often resulting in unaffordable out-of-pocket expenses [43,48,49].

Across the available studies, consistent trends emerged of a substantial burden of untreated caries and periodontal conditions from childhood into adulthood, accompanied by low therapeutic indices and care patterns dominated by pain-driven, extraction-oriented treatment.

These findings highlight key challenges for oral health systems, including persistent inequalities and the difficulty of shifting toward preventive, person-centered models of care. These oral health systems are characterized by a lack of rigorous planning and a predominantly demand-driven approach. Access is severely limited by a marked urban–rural asymmetry, where professionals and services are concentrated in metropolitan areas, leaving rural populations with minimal access to primary care. Furthermore, systemic issues such as poor management and a critical shortage of qualified personnel—as evidenced in countries like Nigeria and Sierra Leone [44,45]—hinder the necessary transition from emergency-driven extractions toward effective, preventive models. Overcoming these structural barriers and high out-of-pocket costs is essential to ensure that oral health is integrated into general health equity. At the policy level, there is an urgent need to integrate oral health into general primary healthcare frameworks and to prioritize the standardization of epidemiological reporting to effectively monitor and address structural inequalities. Furthermore, national health agendas must focus on improving the distribution of the oral health workforce and reducing out-of-pocket costs to ensure access for the most vulnerable populations. Regarding clinical practice, dental professionals and health systems should transition from a reactive, extraction-oriented culture toward preventive and tooth-preserving interventions.

## 5. Limitations

Notwithstanding these constraints, this review provides a comprehensive synthesis of the contemporary oral health landscape in a region characterized by rapid demographic and social transitions. By focusing on evidence published since the late 1990s, the findings offer a reliable baseline for understanding current disease trends and the structural barriers to care. However, the identified gaps in standardized reporting and longitudinal data call for a more harmonized approach in future epidemiological research. The methodology of this study was designed to assess the oral health status of representative national populations across various age groups, noting that studies prior to 1999 were difficult to access and highlighting a lack of research meeting these criteria since 2023. A significant limitation identified in the analyzed evidence is the inconsistent identification and handling of confounding factors, particularly in older descriptive and clinic-based studies. Many of these investigations failed to formally define or systematically adjust for sociodemographic variables in their analyses, which may affect the robustness of the observed associations. To guide future research and improve the quality of evidence for public health policy, it is imperative to adopt harmonized epidemiological approaches that utilize longitudinal designs and standardized reporting metrics, such as Oral Health-Related Quality of Life (OHRQoL).

## 6. Conclusions

These findings highlight a critical paradox in the North and West African regions: while basic oral hygiene is a recognized social norm, the clinical reality is dominated by advanced, untreated pathology and a persistent reliance on emergency extractions. The deep-seated inequalities identified, driven by geography, education, and economic status, underscore that current oral health systems are failing to reach the most vulnerable populations. Consequently, the fundamental challenge for the region lies in dismantling the existing symptom-driven culture.

## Figures and Tables

**Table 1:** Key characteristics of the included studies.

Year	Author (Year)	Study Design	Setting/Data Source	Country	Age of Participants	N	Measurement Instruments	Oral Conditions/Outcomes	SES	Main Findings
1999	Petersen PE et al. [19]	Cross-sectional survey	PB	Niger	6, 12, 18, 35–44 y	1473	WHO-style clinical exam: dmft/DMFT; CPITN; water fluoride	Dental caries and periodontal status	Urbanization: urban/peri-urban/rural Province Sex	56% of 6-year-olds had caries; mean DMFT 1.3 at 12 y and 5.7 at 35–44 y; very high calculus (CPITN 2) in adolescents/adults; caries higher in some rural areas
2000	Blay D et al. [20]	School-based questionnaire survey	SB	Ghana	14–18 y	504	Self-administered questionnaire	Oral hygiene behaviors Sugar consumption	Parents’ education (combined) Urban/rural upbringing	Almost all brushed daily; urban adolescents had higher odds of sugared snack intake and toothpick use; Females and those with more educated parents reported behaviors and sugar intake more often
2002	Bruce I et al. [21]	Cross-sectional clinical survey	PB	Ghana	4–16 y	1851	WHO-style clinical exam: dmft/DMFT, CPITN; dentofacial anomalies	Caries, periodontal conditions, dentofacial anomalies, treatment needs	Peri-urban context; basic socio-demographics (no formal SES index)	Low–moderate caries with many untreated lesions and high gingival bleeding/calculus, indicating poor periodontal health and substantial unmet needs
2004	Abid A [22]	School-based WHO survey	SB	Tunisia	6, 12, 15 y	1802	WHO-style clinical exam: dmft/DMFT, CPITN	Caries, periodontal status, fluorosis, dentofacial anomalies	Geographical workforce inequalities (no individual SES index)	54.4% had caries; DMFT at 12 and 1.3; high need for hygiene instruction and scaling; low therapeutic index with regional workforce maldistribution
2005	Varenne B et al. [23]	Cross-sectional clinical survey	PB	Burkina Faso	15–44 y	14,591	Standardized record form	Presenting dental problems, treatment needs	Occupation Sex Insurance	52.4% attended for pulpal caries; ~60% visits pain-related; strong emergency-driven pattern differing by occupation and insurance
2007	Ojofeitimi EO et al. [24]	Community-based descriptive study	PB	Nigeria	≥55 y	496	Oral Hygiene Index of Greene and Vermillion	Oral hygiene status, chewing problems	Very low schooling (58.1% no formal education)	44.1% chewing difficulties, 29.1% poor/very poor self-rated oral health; poor hygiene and calculus linked to chewing problems and poor self-ratings
2007	Agbelusi GA et al. [25]	Cross-sectional household survey	HB	Nigeria	12 y	1600	WHO-style clinical exam: dmft/DMFT; CPITN. Oral Hygiene Index of Greene and Vermillion	Dental caries (DMFT), oral hygiene status, gingivitis, calculus, malocclusion and other oral conditions; treatment needs	School type (public/private) as proxy for social background; parental occupation; urban LGAs (no formal SES index)	Caries prevalence was 24.6% with mean DMFT 0.46 Oral hygiene was generally fair 73% required periodontal treatment and 35% restorative care
2011	Varenne B et al. [26]	Cross-sectional household survey	HS	Burkina Faso	6–12 y	1606	WHO-style clinical exam: dmft/DMF + Standardized Questionnaires	Caries in primary, permanent, mixed dentitions	Material living index, housing, water, maternal/head education, social networks	Mixed-dentition caries 48.2%; dmft 1.25, DMFT 0.36; higher caries with poor maternal oral status, higher material conditions and low social integration
2016	Agbaje HO et al. [27]	Cross-sectional household survey	HS	Nigeria	1–12 y	983	OHI-S Gingival Index	Oral hygiene, gingivitis	Social class index (mother’s education + father’s occupation)	Age and low SES increased odds of poor hygiene and gingivitis
2019	Oyedele TA et al. [28]	Secondary analysis of cross-sectional school survey	SB	Nigeria	8–16 y	1011	OHI-S	Oral hygiene status	Social class index (mother’s education + father’s occupation)	44.8% good and 17.1% poor hygiene; older age, male sex and low SES reduced odds of good hygiene; last-born children had higher odds of good hygiene
2019	Abbass MMS et al. [29]	Cross-sectional clinical survey	HS	Egypt	3–18 y	369	WHO-style clinical exam: dmft/DMFT; CPITN	Dental caries in primary, mixed and permanent dentitions (dmft, deft, DMFT)	Composite SES (school type/education, guardians’ occupation and address), parental education, BMI, dietary and hygiene behaviors	Overall caries prevalence was 74%, with mean dmft 3.23, deft 4.21 and DMFT 1.04
2020	Aly NM et al. [30]	Cross-sectional household survey	HS	Egypt	6–18 y	392	WHO-style clinical exam: dmft/DMFT; CPITN + OH questionnaire	Caries, oral hygiene, gingival condition	Mother’s education as SES proxy	High caries in primary teeth (67.6%, dft 2.94) and lower in permanent (27.3%, DMFT 0.57); parenting practices not significant but explained variance similar to behaviors
2021	Uguru N et al. [31]	Cross-sectional household survey	HS	Nigeria	Below 20 y 20–40 y 41–60 y Above 60	774	Andersen & Newman-based questionnaire	Caries-related problems, oral care-seeking	Asset-based SES index + food expenditure quintiles	Poorest used traditional/home/PMD care; least poor used private clinics; extractions predominated for caries
2021	Clauss A et al. [32]	Population-based clinical survey	PB	Burkina Faso	15–19 and 35–44 y	827	WHO-style clinical exam: dmft/DMFT; CPITN	Caries, periodontitis	Context of high rural poverty, low workforce	Untreated caries 38% adolescents, 73% adults; DMFT 1.1 and 5.0; attachment loss ≥4 mm in 21% and 61%; very low utilization and fluoride use
2021	Abou El Fadl RK et al. [33]	National population-based cross-sectional study	PB	Egypt	Adults ≥20 y	5954	WHO-style clinical exam: dmft/DMFT; CPITN + Standardized Questionnaires	Periodontitis (CPI ≥ 3), tooth loss not due to caries, gingival bleeding, calculus	Education (illiterate; high school or less; ≥2-year academy/college), urban/rural residence, gender, age; diabetes; smoking, brushing, dental attendance	Periodontitis prevalence 26% (3.2% severe); higher odds in males, illiterate/low-educated adults, smokers and rural residents; Poor oral hygiene, older age, low education and smoking were strong independent predictors of CPI ≥ 3; Tooth loss mainly linked to age, dental attendance, urban residence and diabetes; only 7% had healthy gums and 62% calculus
2022	Diendéré J et al. [34]	WHO STEPS cross-sectional analysis	WS HB	Burkina Faso	25–64 y	4677	WHO STEPS oral questions	Toothbrushing, fluoride use, dental visiting	Education, occupation, urban/rural, marital status	82.8% brushed ≥1/day, 31.4% ≥2/day; ~25% used fluoridated toothpaste; 2.1% visited dentist in 6 months; better practices with higher education, urban residence, female sex
2022	Blankson PK et al. [35]	School-based cross-sectional	SB	Ghana	9–16 y	1118	Clinical exam (DMFT, periodontal disease, trauma, mucosal lesions, malocclusion)	Caries and other oral conditions	Age, sex, previous dental visit	49.7% had ≥1 oral condition; caries 13.3% (mean DMFT 0.27), periodontal disease 30.4%;
2022	Hewlett SA et al. [36]	Population-based cross-sectional survey	PB	Ghana	Adults ≥25 years [3]	729	Clinical examination (soft tissues, tooth count, prosthodontic status, dental caries, periodontal assessment using NHANES/CDC–AAP protocol) Semi-structured questionnaire	Untreated caries, retained roots, gingivitis, periodontitis, tooth loss, oral healthcare coverage	Education, urban/rural residence, district type, health insurance, economic status, oral health service availability	Untreated caries affected about 40% of adults and 26.7% had retained roots, with large variation between districts; metropolitan areas, despite more dentists/clinics and better insurance coverage, showed higher prevalence of missing teeth, retained roots, severe periodontitis and poorer oral healthcare coverage, indicating that service availability alone did not translate into better oral health outcomes
2023	Pengpid S, Peltzer K [37]	WHO STEPS cross-sectional analysis	WS HB	Algeria	18–69 y	6989	WHO STEPS oral questions	Self-rated oral health, OHRQoL, pain, tooth loss, dentures	Education, sex, age, urban/rural etc.	Poor self-rated oral health 37.3%; worse with age, tooth loss, pain, impaired OHRQoL, smokeless tobacco; better with ≥20 teeth, frequent brushing and toothpaste use

Notes: y = year; N = sample size; SES = socioeconomic status indicators; SB = School-based; HB = Household-based; PB = Population-based; WS = WHO STEPS.

**Table 2:** Risk of bias.

Study (Year)	Q1	Q2	Q3	Q4	Q5	Q6	Q7	Q8	Overall Risk of Bias
Petersen PE et al. (1999) [19]	Y	Y	Y	Y	U	U	Y	Y	Moderate
Blay D et al. (2000) [20]	Y	Y	Y	U	Y	Y	Y	Y	Low
Bruce I et al. (2002) [21]	Y	Y	Y	Y	U	U	Y	Y	Moderate
Abid A (2004) [22]	Y	Y	Y	Y	U	U	Y	Y	Moderate
Varenne B et al. (2005) [23]	Y	Y	U	Y	U	U	Y	Y	Moderate
Ojofeitimi EO et al. (2007) [24]	Y	Y	Y	Y	U	U	Y	Y	Moderate
Agbelusi GA al. (2007) [25]	Y	Y	Y	Y	U	U	Y	Y	Moderate
Varenne B et al. (2011) [26]	Y	Y	Y	Y	Y	Y	Y	Y	Low
Agbaje HO et al. (2016) [27]	Y	Y	Y	Y	Y	Y	Y	Y	Low
Oyedele TA et al. (2019) [28]	Y	Y	Y	Y	Y	Y	Y	Y	Low
Abbass MMS et al. 2019) 2019 [29]	Y	Y	Y	Y	U	U	Y	Y	Moderate
Aly NM et al. (2020) [30]	Y	Y	Y	Y	Y	Y	Y	Y	Low
Uguru N et al. (2021) [31]	Y	Y	Y	Y	Y	Y	Y	Y	Low
Clauss A et al. (2021) [32]	Y	Y	Y	Y	U	U	Y	Y	Moderate
Abou El Fadl RK et al. (2021) [33]	Y	Y	Y	Y	Y	Y	Y	Y	Low
Diendéré J et al. (2022) [34]	Y	Y	Y	Y	Y	Y	Y	Y	Low
Blankson PK et al. (2022) [35]	Y	Y	Y	Y	U	U	Y	Y	Moderate
Hewlett SA et al. (2022) [36]	Y	Y	Y	Y	Y	Y	Y	Y	Low
Pengpid S, Peltzer K (2023) [37]	Y	Y	Y	Y	Y	Y	Y	Y	Low

Y = Yes; N = No; U = Unclear.

## Data Availability

No new data were created or analyzed in this study. Data sharing is not applicable to this article.

## Supplementary Materials

The following supporting information can be downloaded at: https://www.mdpi.com/article/10.3390/healthcare14060821/s1, Supplementary File S1: PRISMA checklist; Supplementary File S2: Database search strategy.
