# Supplementary material for: Oral Health Trends and Challenges in North and West Africa: A Systematic Review of Cross-Sectional Studies
Source: Healthcare (Basel). 2026 Mar 23;14(6):821. doi: 10.3390/healthcare14060821 (PMC13026218; doi:10.3390/healthcare14060821)
Supplement: Supplementary file 1 [file healthcare-14-00821-s001.zip › healthcare-4162429-supplementary file S2.pdf]

Database search strategy

**Pubmed**	*("Argelia"[All Fields] OR "Benin"[MeSH Terms] OR "Burkina Faso"[MeSH Terms] OR "Egypt"[MeSH Terms] OR "Cabo Verde"[MeSH Terms] OR "Costa de Marfil"[All Fields] OR "Gambia"[MeSH Terms] OR "Ghana"[MeSH Terms] OR "Guinea"[All Fields] OR "Guinea-Bissau"[All Fields] OR "Equatorial Guinea"[MeSH Terms] OR "Liberia"[MeSH Terms] OR "Libya"[MeSH Terms] OR "Mali"[MeSH Terms] OR "Morocco"[MeSH Terms] OR "Mauritania"[MeSH Terms] OR "Niger"[MeSH Terms] OR "Nigeria"[MeSH Terms] OR "Senegal"[MeSH Terms] OR "Sierra Leone"[MeSH Terms] OR "Sudan"[MeSH Terms] OR "Togo"[MeSH Terms] OR "Tunisia"[MeSH Terms]) AND ("oral health"[MeSH Terms] OR "dental caries"[MeSH Terms] OR "periodontal diseases"[MeSH Terms] OR "tooth loss"[MeSH Terms])*	995
**Scopus**	*(INDEXTERMS("Algeria") OR INDEXTERMS("Benin") OR INDEXTERMS("Burkina Faso") OR INDEXTERMS("Egypt") OR INDEXTERMS("Cape Verde") OR INDEXTERMS("Ivory Coast") OR INDEXTERMS("Gambia") OR INDEXTERMS("Ghana") OR INDEXTERMS("Guinea") OR INDEXTERMS("Guinea-Bissau") OR INDEXTERMS("Equatorial Guinea") OR INDEXTERMS("Liberia") OR INDEXTERMS("Libya") OR INDEXTERMS("Mali") OR INDEXTERMS("Morocco") OR INDEXTERMS("Mauritania") OR INDEXTERMS("Niger") OR INDEXTERMS("Nigeria") OR INDEXTERMS("Senegal") OR INDEXTERMS("Sierra Leone") OR INDEXTERMS("Sudan") OR INDEXTERMS("Togo") OR INDEXTERMS("Tunisia")) AND (INDEXTERMS("oral health") OR INDEXTERMS("dental caries") OR INDEXTERMS("periodontal diseases") OR INDEXTERMS("tooth loss"))*	936
**WoS**	*("Algeria" OR "Benin" OR "Burkina Faso" OR "Egypt" OR "Cape Verde" OR "Ivory Coast" OR "Gambia" OR "Ghana" OR "Guinea" OR "Guinea-Bissau" OR "Equatorial Guinea" OR "Liberia" OR "Libya" OR "Mali" OR "Morocco" OR "Mauritania" OR "Niger" OR "Nigeria" OR "Senegal" OR "Sierra Leone" OR "Sudan" OR "Togo" OR "Tunisia") AND ("oral health" OR "dental caries" OR "periodontal diseases" OR "tooth loss")*	1,419

Note: WoS=Web of Science
